# Assessment of the Potential Role of Tryptophan as the Precursor of Serotonin and Melatonin for the Aged Sleep-wake Cycle and Immune Function: *Streptopelia Risoria* as a Model

**DOI:** 10.4137/ijtr.s1129

**Published:** 2009-01-14

**Authors:** Sergio D. Paredes, Carmen Barriga, Russel J. Reiter, Ana B. Rodríguez

**Affiliations:** 1 Department of Physiology (Neuroimmunophysiology Research Group), Faculty of Science, University of Extremadura, Badajoz, Spain; 2 Department of Cellular and Structural Biology, University of Texas Health Science Center, San Antonio, Texas, U.S.A

**Keywords:** immune function, melatonin, serotonin, sleep-wake cycle, ringdove, tryptophan

## Abstract

In the present review we summarize the relationship between the amino acid, tryptophan, the neurotransmitter, serotonin, and the indole, melatonin, with the rhythms of sleep/wake and the immune response along with the possible connections between the alterations in these rhythms due to aging and the so-called “serotonin and melatonin deficiency state.” The decrease associated with aging of the brain and circulating levels of serotonin and melatonin seemingly contributes to the alterations of both the sleep/wake cycle and the immune response that typically accompany old age. The supplemental administration of tryptophan, e.g. the inclusion of tryptophan-enriched food in the diet, might help to remediate these age-related alterations due to its capacity of raise the serotonin and melatonin levels in the brain and blood. Herein, we also summarize a set of studies related to the potential role that tryptophan, and its derived product melatonin, may play in the restoration of the aged circadian rhythms of sleep/wake and immune response, taking the ringdove (*Streptopelia* *risoria*) as a suitable model.

## Introduction

Tryptophan is a polar, hydrophobic amino acid indispensable for protein synthesis. It is classified as an “essential” amino acid, i.e. it cannot be synthesized by the human organism and must therefore be ingested in the diet. Once tryptophan is consumed, it is readily absorbed into the capillaries in the intestinal wall. A small amount of the amino acid remains free while the majority of it (roughly 80%–90%) is transported bound to albumin through the blood and into the brain. This transport may be altered by the competition exerted by other free, neutral amino acids of high molecular weight, branched-chain amino acids, including valine, leucine and isoleucine, as well as phenylalanine and tyrosine, which bind to the same transporters.^1^,^2^

**Figure 1.**
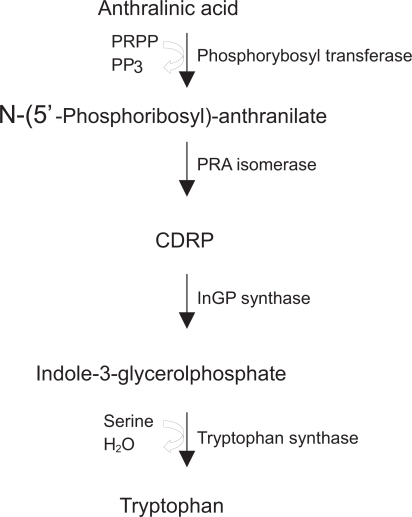
Scheme of the conversion of anthranilic acid to tryptophan. PRPP, phosphoribosylpyrophosphate; PP_3_, pyrophosphate; PRA, N-(5’Phosphoribosyl)-anthranilate; CDRP, 1-(o-Carboxyphenylamino)-1-deoxyribulose-5-phosphate; InGP, indole-3-glycerolphosphate.

The metabolism of tryptophan is complex. It is involved in a variety of metabolic pathways and requires a suitable quantity of biopterin, magnesium or vitamin B6, which is involved in the conversion of the amino acid into serotonin and in the metabolism of other by-products, such as kynurenine. The main precursor of tryptophan is the anthralinic acid or anthranilate, a compound that after a series of chemical reactions is transformed into indole-3-glycerolphosphate. The enzyme tryptophan synthase converts this latter compound into glyceraldehyde 3-phosphate in a two-step reaction with the intermediary indole bound to the active site of the enzyme and with the intervention of serine (Fig. 1). The contribution of tryptophan to energetic metabolism is double since on one hand it is ketogenic, i.e. it forms acetyl coenzyme A, and on the other it is glucogenic, as it produces alanine.^3^

Tryptophan is transported to the liver where it is metabolized. Thereafter, in consecutive reactions it is transformed into nicotinic acid and other subproducts that either are stored or serve as a basis for important substances including quinolinate, picolinate or glutarate.^4^

**Figure 2.**
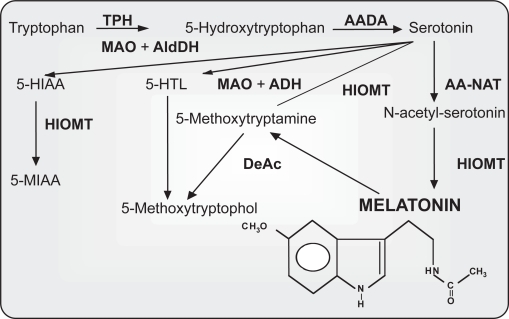
Pathways of indole metabolism in photosensitive pineal cells. Enzymes: AADA, aromatic L-amino acid decarboxylase; AA-NAT, aralkylamine N-acetyltransferase; DeAc, deacetylase; HIOMT, hydroxyindole-O-methyltransferase; MAO, monoamine oxidase; TPH, tryptophan hydroxylase. Indoles: N-acetyl-serotonin; 5-HIAA, 5-Hydroxyindoleacetic acid; 5-HTL, 5-hydroxytryptophol; 5-MIAA, 5-methoxyindole-3-acetic. Chemical structure of melatonin is shown at the bottom of the figure (taken from Paredes, 2007,^178^ modified).

The amino acid tryptophan is the precursor of several important products including serotonin or melatonin (Fig. 2). These molecules are biogenic amines of low molecular weight that belong to the indole group. It has been observed that the synthesis of melatonin in the pineal gland diminishes with aging.^5^–^7^ This is believed to be due to degenerative changes in the neural structures (postganglionic neurons) innervating the pineal gland and central nervous system, rather than to the degeneration of the pineal tissue *per se*,^8^ as well as to a reduction in the quantity of the necessary precursor, serotonin.^7^,^9^,^10^ However, the number or sensitivity of melatonin receptors throughout the organism may decline with age as a result of the usual degenerative processes.^11^ The result is the development of the so-called “serotonin and melatonin deficiency state.”^12^–^14^ This age-related state seemingly contributes to the alterations of both the sleep-wake cycle and the immune responsivity that characterizes aging. Thus, the consumption of tryptophan as a pharmacological agent or as part of a diet rich in this amino acid may attenuate age-related changes in circadian organization, the immune system, sleep, and other disorders, due to its ability to elevate circulating levels of serotonin and melatonin.^15^–^18^

This review is constructed to provide a concise view of the effects of tryptophan, serotonin and melatonin on the sleep-wake cycle and the immune system responses, to identify possible links between the impairment of these rhythms and the reduction in serotonin and melatonin levels in the aging organism, and to illustrate the potential restorative role that tryptophan may play against the age-related afore-mentioned circadian alterations. Finally, we present the ring dove (*Streptopelia risoria*) as a suitable model for the study of the aged sleep-wake or activity-rest rhythms and immunosenescence and summarize the results obtained in this animal species in the circadian rhythm research field over the last decade.

## Tryptophan, Serotonin, Melatonin, and the Sleep-wake Cycle

The initiation of investigations related to the hypnotic effects that tryptophan exerts on the human sleep dates back to the 70s and 80s,^19^,^20^ when it was observed that this amino acid augmented the propensity to sleep. During this interval tryptophan was used as a successful therapeutic agent to combat chronic insomnia.^21^ More recently, the consolidation properties of tryptophan for the sleep-wake rhythm of newborns have been tested. It has been shown that being fed with nutritionally dissociated milk formulas, i.e. a diurnal formula with low content of tryptophan and carbohydrates and a high amount of protein, supplemented with the nucleotides cytidine 5-monophosphate, guanosine 5-monophosphate and inosine 5-monophosphate, and a nocturnal formula that contains high levels of tryptophan and carbohydrates, a low level of protein, with the nucleotides adenosine 5-monophosphate and uridine 5-monophosphate, improved the total hours of sleep, the efficiency of sleep, the minutes of nocturnal immobility, and reduced both the number of nocturnal awakenings and the sleep latency of newborns.^22^,^23^ This is of special importance at a stage of life where an appropriate rest period is directly related to an optimal development of both the nervous and immune systems.

The first studies showing a relationship between serotonin and sleep appeared in the middle of the 50s. It was observed that reserpine, an antipsychotic, antihypertensive indole alkaloid dimin ished the concentration of serotonin in the brain and induced a sedative state analogous to sleep.^24^ It was also reported that the parenteral injection of L-5-hydroxytryptamine caused cortical synchronization and that inhibitors of the enzyme monoamine oxidase selectively suppressed paradoxical sleep or REM for long periods of time that could persist for days or even weeks.^25^ Serotonin subsequently appeared as a key factor in understanding some of the mechanisms involved in the sleep-wake cycle. In fact, subsequent investigations documented that the destruction of the raphe nuclei, an area with an abundance of serotonin-containing neurons, by means of coagulation, produced insomnia for lengthy periods of time (10–15 days). Thus, a relationship among insomnia intensity, the magnitude of the injury in the raphe nuclei and the amount of brain serotonin that remained in the telencephalon after the degeneration of the serotoninergic terminals was established. Particularly, a correlation between the destruction of the rostral raphe and the slow-wake sleep and the telencephalic serotonin, which decreased, and between paradoxical sleep and damage to the nucleus raphe magnus was identified.^26^,^27^ It was additionally pointed out that p-chlorophenylalanine inhibited the enzyme tryptophan hydroxylase, which in turn impaired the biosynthesis of serotonin and secondarily led to states of total insomnia.^28^

These experiments led to the elaboration of the so-called “monoaminergic theory of sleep.” This established that serotonin, or somnotonin as it was named by Koella,^29^ was the neurotransmitter or “neurohormone” of sleep, since it produced sleep by the inhibition of the reticular formation and locus ceruleus, the putative centers of wakefulness. Conversely, catecholamines were found to be responsible for awakening. The demonstration, however, that the electric activity of serotoninergic neurons as well as the release of serotonin increased during wakefulness and decreased with sleep was seemingly in clear contradiction with the afore-mentioned theory. In the late 80s, however, a relationship between the sleep/wake cycle and serotonin was again considered. More recent experiments suggest that during wakefulness, serotonin is responsible for initiating a cascade of post-synaptic genomic processes in hypnogenic neurons located in the pre-optic area.^30^ Through these processes, the release of the neurotransmitter during wakefulness leads to a homeostatic regulation of slow wave sleep,^30^ also acting as a positive modulator of melatonin synthesis.^31^

Among the diverse physiological functions in which melatonin has been involved, its role as regulator of the sleep/wake rhythms has attracted the attention of a number of sleep researchers in the last decade. The discovery that melatonin was mainly secreted at night and the tight relationship between the nocturnal increase of endogenous melatonin and the existing co-ordination of sleep as well as the pro-somnogenic effects that the pineal indole seemingly possessed, led many investigators to suggest that melatonin was likely implicated in the physiological regulation of sleep. With regard to this presumption, it was observed that the suppression of the production of melatonin using β-blockers correlated with insomnia,^32^–^34^ while the increase of the plasma levels of melatonin by reducing the activity of the enzymes that metabolize the indole in the liver resulted in an augmentation of the somnolence state.^35^

It was reported that during the wake period immediately prior to sleep, known as the wake-maintenance zone or “forbidden zone” for sleep,^36^ the propensity for sleep is reduced to a minimum and, at the same time, the activity of the neurons of the central nervous system is elevated.^37^,^38^ Thus, the transition from the wake stage to a period of high propensity for sleep coincides with the nocturnal elevation of the endogenous rhythm of melatonin.^39^ This increase seems to be temporally related to the opening of the so-called sleep gate.^40^,^41^

Taking into account the relationship between the endogenous secretion of melatonin and the opening of the entry into nocturnal sleep, it has been proposed that the role of the pineal indole does not involve an active induction of sleep, rather it consists of the inhibition of the mechanisms that generate the circadian period of wakefulness,^42^ presumably through the MT1 melatonin receptor^43^,^44^ and GABAergic activation^45^,^46^ at the central nervous system level.

Regarding the effects of the exogenous administration of melatonin on sleep and the circadian clock, there are a number of studies reporting that diurnal treatment with the indole produces drowsiness, 47–51 as well as raising the circulating levels of melatonin to values normally observed at night.^52^

For these reasons, melatonin, through its actions in the central nervous system, is seemingly a crucial substance for the co-ordination of the circadian mechanism of sleep. However, the recent discovery of melatonin receptors in other brain areas such as the hippocampus^53^ makes necessary further investigation to elucidate the exact role of melatonin on sleep in the different brain structures. Moreover, considering the rather high levels of melatonin in certain plant foodstuffs (Table 1),^54^ the consumption of melatonin through the diet may have significant benefits to human and animal health.

## Tryptophan, Serotonin, Melatonin, and the Immune System

The concentration of the amino acid tryptophan is lower in psychologically depressed patients with respect to control individuals.^55^ This consequently produces a decrease in the levels of serotonin, a neurotransmitter that has frequently been implicated in depressive syndromes.^56^ Moreover, it has been observed that when depressive disorders appear, they are accompanied by an inflammatory response involving the immune system, which is inversely proportional to the concentration of tryptophan in plasma.^57^ This is also negatively correlated to the number of leukocytes and other components of the immune system including interleukin 6 (IL-6) and IL-8.^58^ It has been reported that individuals with sleep disturbances experience the same symptoms as patients suffering from depression, i.e. a diminution of the tryptophan levels in plasma and an augmentation of both IL-6 and IL-8, compared to healthy individuals.^56^,^57^ They also experience a decrease in the levels of IL-2.^59^ On the other hand, when interruption of sleep for 5 hours during the nocturnal period occurs, the levels of IL-1 and IL-2 are elevated. When somnolence is produced in excess, IL-6 and tumor necrosis factor α (TNF-α) are elevated,^60^ with a subsequent rise in the number of monocytes and neutorphils.^61^

Regarding the effect that the amino acid exerts on the phagocytic function, recent studies suggest an enhancement of phagocytosis after the oral administration of tryptophan. Particularly, it has been observed that administering the amino acid to rats causes incremental changes in circulating levels of melatonin as well as stimulating the antigenic capacity of ingestion of peritoneal macrophages obtained during the nocturnal period.^15^,^62^ An elevation of the phagocytic capacity at night has also been observed in otherwise untreated rats and mice.^63^–^66^ This suggests that the activation of the innate immune response after tryptophan consumption may be due to its conversion into the pineal indole. In fact, it has been shown that macrophages obtained from the peritoneal cavity of normal rats when incubated with tryptophan show an increase in arylalkylamine N-acetyltransferase activity which corresponds to a rise in melatonin production.^67^ Nevertheless, tryptophan is also the precursor of serotonin, a compound that may also play a role in the function of the innate immune system. Owing to the fact that receptors for serotonin exist in leukocytes and a transporter for this amine has been found in macrophages, mononuclear leukocytes, and B cells, this neurotransmitter may be a critical element for the connection between the nervous and immune systems.^68^,^69^ Some studies have shown that serotonin may also possess an antioxidative role.^70^–^72^ Serotonin has also been reported to inhibit leukocyte phagocytosis,^73^,^74^ especially when the concentrations of neurotransmitter used are in the pharmacological range.^71^ Since circadian variations of serotonin in plasma and different brain regions have been observed,^75^ this may somehow influence the circadian daily variations of the immune system.

A substantial body of research has defined melatonin as a remarkable molecule with pleiotropic effects both of an endocrine and a non-endocrine nature on the immune system.^76^–^78^ The abolition of the daily rhythm of melatonin via either surgical or functional pinealectomy has been shown to directly correlate to weight loss of the thymus as well as to the abnormal involution of this immune organ; this is also accompanied by a depletion of lymphoblasts and an almost total absence of lymphocytes. 79 A reduction in the size of lymph nodes associated with follicular loss in the outer cortex^80^ together with an alteration of the activities of thymic polyamine biosynthetic amines have also been noted.^81^–^83^ Other immune organs such as the spleen or the bursa of Fabricius in birds are impaired following pinealectomy. In this respect, Brainard et al.^79^ showed a lack of evident germinal centers and an apparent inactivity of the red pulp in the Syrian hamster spleen, while Jankovic et al.^84^ found a delayed development not only in the bursa but also in the thymus and spleen of pinealectomized chicks. The absence of the pineal gland has also been reported to significantly reduce IL-2 production and NK activity^85^,^86^ and decrease the cellular and humoral immune response of both mammals and birds.^87^–^90^ When melatonin is administered to pinealectomized animals, the effects on immune system are typically reversed.

*In vivo* models have shown melatonin to be considered as a positive regulator of immune responses. The administration of melatonin results in the enhancement of antigen presentation by splenic macrophages in major histocompatibility complex II, IL-1 and TNF-α production,^91^ the increase in the generation of thymosin α1 through a rise in prothymosin α gene expression 92 as well as the production of IL-10.^93^ In mice, treatment with melatonin also upregulates macrophage-colony stimulating factor, TNF-α, transforming growth factor β and stem cell factor gene expression in peritoneal macrophages and the levels of IL-1β, interferon γ, macrophage-colony stimulating factor, TNF-α and stem cell factor in splenocytes.^94^

The pineal indole also possesses potential positive effects on several immune system pathologies including acute and chronic inflammation^95^,^96^ and syndromes provoked by certain viruses such as the encephalomyocarditis virus,^97^ lethal Semliki Forest virus and the attenuated non-invasive West Nile virus^98^ as well as the Venezuelan equine encephalomyelitis virus.^99^–^101^

## The Serotonin and Melatonin Deficiency State Due to Ageing: Effects and Consequences on the Sleep/wake Cycle and the Immune System

Aging is associated with a reduction in the size of the brain. These changes are generally attributed to a loss of neurons in specific layers and regions of the brain, although there exists considerable interindividual variation.^102^ The loss of neurons has been shown to occur in the locus ceruleus, the main source of catecholaminergic neurons, and in the substantia nigra, where dopaminergic neurons are most abundant. This may contribute to age-related changes in homeostasis, sleep alterations, stability, movement, and cognitive function.^103^ Alterations tend to affect the myelinated axons (the white matter) at a much greater degree when compared to the neuron cell bodies in the grey matter.^104^ Aging has also been proposed to modify the permeability of the blood-brain barrier, which may have consequences in terms of porosity of this structure to different drugs or molecules and to cause a decline in the brain metabolism and blood flow.^105^

Neurotransmitter functions of serotonin are widely distributed in the central nervous system and are related to the regulation of a variety of behaviors. Serotonin is seemingly involved in the regulation of humor, anxiety, sleep, appetite, sexual function, brain blood flow and many other functions. The serotoninergic neurons are located in the raphe nuclei of the brain stem and their axons project to all brain areas, including the cerebral cortex, thalamus, the limbic system and the hypo-thalamus. In regard to this, any change in the number of serotonin receptors or in the endogenous levels of the neurotransmitter due to aging may have consequences on behaviour or cognitive function. Diverse studies have shown that alterations in serotinergic neurotransmission cause age-related alterations. Particularly, a reduction in the density of the serotonin type 2 receptor (5HT2A) has been described.^106^

The injection of altanserin, a high affinity ligand to 5HT2A receptor union sites, to young and old subjects showed that the specific union for this receptor was significantly reduced in old individuals compared to the young, as well as the number of receptors, whose loss was marked in certain brain regions.^106^ Since many antidepressant drugs typically relieve the symptoms of depression by blocking serotonin reuptake in order to facilitate an increase in serotonin activity, it is speculated that the high incidence of depression in the elderly may be attributed to the reduction in serotonin receptors. Moreover, it has been reported that serotonin receptors and transporters are less sensitive to hormone regulation, which responds to the deficiency associated to aging of the regulation of the hippocampal serotoningeric system exerted by corticosterone.^107^ This suggests that the age-related changes in the neurochemistry of serotonin may be a cause of the increased rates of depression and hypercortisolemia observed in the aged populations. In parallel, serotonin is known to increase the quality of slow wave sleep,^30^ as well as being a waking neurotransmitter.^108^ In addition, the close relationship between serotonergic activity and the adjustments of circadian phase has suggested that serotonin also plays a role in the endogenous regulation of the circadian clock.^109^ These findings point to the participation of serotonin neurotransmission in the behavioral alterations commonly observed in aged individuals and may have potential therapeutic implications.

**Figure 3.**
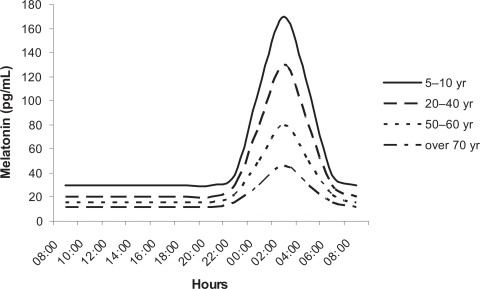
Diagrammatic representation of daily profiles of serum melatonin levels throughout lifespan (taken from Karasek and Reiter,^13^ modified).

Blood melatonin levels show a clear circadian rhythm, with low levels during the day and high values at night, with these values being 10/15-fold greater that those measured in the diurnal period.^110^,^111^ In humans, the indole has been shown to gradually decrease during the increased life span, with the day/night rhythm being practically absent in individuals over 65-yr old (Fig. 3).^112^ This observation has also been reported when melatonin levels in young and old rats, gerbils, hamsters or ringdoves are compared.^113^,^114^ It is believed that the amplitude of the nocturnal melatonin rhythm is genetically determined as it shows important interindividual differences,^115^ even though in a given individual it exhibits a high degree of fidelity over time.^116^ Hence, some subjects produce significantly less melatonin in their life than others, which may be of importance for aging.^13^

Aging is a crucial factor in terms of sleep characteristics. The structure, depth, and continuity of sleep tend to change over the life span.^117^ Some reports have shown that more than a third of the elderly experience recurrent difficulty to maintain sleep^118^ due to impairment in both the quality and the quantity of sleep.^119^ Sleep onset latency usually increases together with the number and duration of awakenings, while sleep stability declines, and sleep consolidation is altered.^117^ It is therefore not surprising that the information provided by epidemiologic studies reveals that up to 40% of individuals over 65-yr old complain about sleep problems and 12%–25% suffer from persistent insomnia.^120^ The number of elderly people that have been prescribed sleep drugs or that use aids to facilitate nocturnal rest is estimated to be around 14%.^121^

Aging also causes alterations in the amplitude of the sleep/wake circadian rhythm.^122^ The temporal organization of sleep is impaired and the regulatory mechanisms of the sleep processes are attenuated.^123^ Several reasons suggested for these age-related changes are a reduction in the number of pinealocytes, changes in retina and in the suprachiasmatic nuclei or alterations in melatonin secretion.^124^

The effectiveness of the immune system decreases during aging. The lymphoid tissues of the spleen, bone marrow and thymus are progressively lost; this increases the incidence of infections, autoimmune diseases and cancer.^125^ With advancing age, the number and proliferative capacity in response to mitogen-stimulation of the diverse subpopulations of T-lymphocytes is reduced^126^,^127^ while apoptosis is elevated.^128^ Moreover, the synthesis and secretion of immunoglobulins is delayed, presumably due to a lack of appropriate levels of cytokines;^129^ this decreases the competence of antibodies in immunization against infectious agents.^130^ Antigen-presenting cells or accessory cells and phagocytes experience an age-related rise in the oxidative state,^131^ resulting in a reduced capability to adapt to environmental stress and in a reduction of the phagocytic parameters.^114^

It is known that the pineal gland influences the function of the neuroendocrine system and the efficacy of the immune system to recognize and react to any endogenous or exogenous factor.^132^ For this reason, it has been suggested that aging is a result of the deterioration of this key factor of the pineal gland due to a deficient melatonin secretion and a decline in the melatonin/serotonin ratio. This may impair several aspects of an individual’s neurophysiology. 48 It has been observed that early extirpation of the pineal gland produces substantial accumulations of lipid peroxidation products, oxidized DNA, reduced fluidity of cell membranes and elevated protein damage in many organs.^133^ These changes are a consequence of the loss of the endogenous melatonin rhythm. Impairment in melatonin synthesis is thought to likely play a role in the aging process since this indole participates in vital defence mechanisms including free radical scavenging and indirect antioxidative actions.^134^,^135^ Thus, melatonin is estimated to be responsible for the scavenging of ten or more reactive damaging agents.^136^ Furthermore, its initial, secondary, tertiary and quaternary derivatives are all potent scavengers that, together with melatonin, form a remarkable cascade of reactions referred to as melatonin’s antioxidative cascade.^136^,^137^ The detoxification of radical and radical products by melatonin and its derivatives are receptor-independent actions and only require that the scavenger be at the site where the radical product is generated.^138^ This is essential since highly reactive agents mediate damage in the immediate vicinity of where they are produced, i.e. the damage is site specific.^138^ Melatonin also has receptor-mediated actions which adds to the capability of this molecule in eradicating radicals and reducing oxidative stress.^139^,^140^ Thus, melatonin stimulates a number of antioxidative enzymes which metabolize reactive products to innocuous agents. The enzymes whose activities have been shown to be promoted by melatonin include both Cu/Zn and Mn superoxide dismutases, glutathione peroxidases and glutathione reductase.^139^,^141^,^142^ The effects of melatonin on the activities of the antioxidative enzymes are likely receptor-mediated and involve receptors on the plasma membrane and also presumably receptors/binding sites in the nucleus.^140^

The efficiency of sleep is also reduced as a result of low circulating levels of the pineal indole. These phenomena typically accompany advancing age. Melatonin may thus protect against the oxidation of essential molecules,^143^,^144^ which appear in significant numbers in aged organisms,^145^ and resist neurodegenerative disorders associated with the impairment produced in particular brain areas by free radicals.^146^ In fact, melatonin may possess benefits in Parkinson and Alzheimer diseases.^147^,^148^

Pinealectomy is believed to accelerate the aging process, causing high blood pressure, elevated alkaline phosphatase activity, modification in the synthesis of prostaglandins, and induction of REM. These alterations are seemingly counteracted by the administration of melatonin.^149^ Many studies support the idea that melatonin may be considered as an anti-aging and rejuvenating product. The evidence accumulated to date supports the hypothesis that the supplemental treatment with melatonin may be of benefit during aging.^150^,^151^

## The Potential Restorative Role of Tryptophan of the Impaired in the Sleep/wake Cycle and Immune System that Accompany Aging: *Streptopelia Risoria* as a model

The ringdove (*Sterptopelia risoria*) is a species characterized by being diurnal and monophasic with sleep-wake cycles similar to those of human beings and, therefore, it represents a good model to investigate impairments in the circadian system due to age, including immune alterations.

The first study performed in the ringdove that showed a relationship between the pineal gland, melatonin and the immune system was that of Rodríguez and Lea.^87^ Pinealectomy produced a significant increase in the number of total white blood cells and total protein concentration in plasma in addition to altering different stages of the phagocytic process. Also, during an immunization study, a reduction in the percentage of leukocytes and lymphocytes and an increase in the percentage of heterophils accompanied by a rise in the concentration of serum corticosterone were observed 3 hr following treatment. For the immunological parameters, adherence capacity and latex bead ingestion were increased 3 hr after normal sleep serum injection and the nitroblue tetrazolium reduction test 3 and 24 hr after normal sleep serum treatment. In addition, the administration of normal sleep serum produced a significant increase in serum T3 and T4 concentrations 4 days following injection. These results indicate that the loss of melatonin due to pinealectomy has a marked effect on both the number and function of immune cells.

In reference to *in vivo* experiments with melatonin, a correlation between the circadian rhythm of the indole, phagocytosis, and superoxide anion levels has been reported.^152^ Thus, the elevated melatonin serum levels during the dark period coincide with an enhanced phagocytosis of inert particles and lower superoxide anion levels derived from the immune system. These effects where reinforced when the animals received melatonin orally, which also elevated circulating levels of the indole.^153^,^154^ Similar results were observed when the phagocytosed particle was a living organism (*Candida albicans*) with the effect being dose-dependent.^153^,^155^ *In vitro* experiments have reported similar results, with the chemoattractant ability for heterophils being significantly enhanced by the pineal indole^156^ as well as an augmentation of the phagocytic function and a decline in the free radical production.^65^ Melatonin also decreased the superoxide dismutase activity (an indicator of the metabolic burst) in heterophils after the ingestion of latex beads.^157^

The concentration of malonaldehyde in cells is an index of induced oxidative damage to membrane lipids. The co-incubation of a heterophil suspension with or without inert particles (latex beads), as material to be phagocytosed, in combination with melatonin has been shown to clearly reduce the production of malonaldehyde. The enhancement of malonaldehyde levels produced by latex beads was also annulled in the samples incubated with melatonin.^158^

In stressful situations, an alteration in the endogenous circadian rhythm of melatonin in the ringdove has been described.^159^ This has also been reported in mammals, where a decreased MESOR and amplitude of the melatonin rhythm, and a significantly elevated MESOR of the corticosterone rhythm have been observed.^66^,^160^

*Streptopelia risoria* also experiences a “serotonin and melatonin deficiency state” during aging;^7^,^10^ this is associated with increased nocturnal activity and depressed immune function.^7^,^154^,^161^,^162^ Under these conditions, orally administered melatonin has been reported to improve nocturnal rest not only in old ringdoves, but also in young birds.^161^,^162^ This is likely to be a result of a decrease in the core temperature and an increase in the peripheral temperature observed after the oral administration of the indole in this species.^162^ Thus, melatonin may be used to palliate the reduction in the thermoregulatory responses and the capacity for thermal comfort reported in the elderly.^163^ This is of importance since sleep disorders are believed to be caused, at least partly, by changes in the circadian rhythms of temperature and melatonin.^164^

The oral administration of the indole restores some of the changes that aging produces in the innate immune response, with an enhancement in the phagocytic processes and a decrease in the production of free radicals, reflecting the scavenging properties of melatonin; this is most probably due to the restoration of the nocturnal rise of circulating melatonin due to its administration.^153^,^154^,^161^,^165^ This hypothesis is corroborated by previous findings showing that the incubation of ringdove heterophils obtained from old animals with the physiological concentrations of serum melatonin typical of young and mature birds induced a dose-dependent rise in both the phagocytic index and the candidicide capacity, together with a decline in superoxide anion levels.^166^ Furthermore, the incubation of old heterophils with the physiological concentrations of melatonin characteristic of young animals (50 and 300 pg/ml, diurnal and nocturnal, respectively) counteracted the enhancement of malonaldehyde levels caused by latex beads, with the effect being greater at the longer incubation time tested.^167^

Once the potential role of the pineal indole to reverse the age-related alteration in the activity/rest rhythms and immune impairment in the ringdove was documented, the next step was to test whether tryptophan, the precursor of melatonin and also of the neurotransmitter serotonin would have similar effects. Tryptophan administered in the diet is known to increase the availability of serotonin in the brain, improve the EEG delta potential, and elevate the amount of NREM.^168^ It has also been observed in mammals that orally ingested tryptophan increases brain levels of serotonin during the day and the circulating levels of melatonin during the immediately subsequent night.^62^ Likewise, tryptophan administration raised the circulating levels of both serotonin and melatonin in rats.^15^,^18^ In sexually immature ringdoves, the administration of the amino acid increased nocturnal rest, which seemingly correlated with the augmented circulating levels of melatonin caused by tryptophan treatment.^169^ Tryptophan significantly increases the hippocampal, striatal, and hypothalamic serotonin contents,^10^ and reduces the expression of c-fos in the suprachiasmatic nucleus.^170^ C-fos levels are high in several cerebral regions during spontaneous waking or sleep deprivation and fall after a few hours of sleep.^171^

In old ringdoves, the treatment with 300 mg of tryptophan per kg b.w. reduces nocturnal activity without affecting their diurnal activity, an effect accompanied by a general increase of serum serotonin levels^16^ This increase of serum serotonin indicates a higher availability of tryptophan which, after passing the blood–brain barrier, would be converted into serotonin.^10^,^15^,^18^,^31^,^62^ The elevated serotonin in the pineal gland serves as a substrate for melatonin synthesis, and increases in the levels of this molecule would reduce nocturnal activity of old ringdoves,^16^ improving their aging-impaired nocturnal rest.^7^ Tryptophan administered at the same dose and time also provoked an improvement of the circadian rhythm of temperature in this species.^162^

Regarding the innate immune response in old birds, treatment with tryptophan produced a significant diurnal and nocturnal augmentation in phagocytic parameters; the values reached during the night were significantly higher that those measured during the day.^17^ This is consistent with earlier findings demonstrating that giving the amino acid to mammals^15^,^62^ or birds,^172^ or melatonin itself to these species^14^,^154^,^165^,^173^,^174^ has a general immuno-enhancing effect. Moreover, a reduced production of superoxide anion radicals in old ringdoves was observed after tryptophan treatment.^17^ This change was presumably due to the rise of the circulating serum levels of melatonin produced by the exogenous administration of its precursor.^16^,^17^ This indicates that rising serum levels of melatonin are accompanied by a decline in the levels of superoxide anion radicals produced by heterophils, as reported previously. Also, tryptophan significantly limits the reduction in cell viability of heterophils exposed to hydrogen peroxide. 17 A similar effect was obtained when the cells are incubated with melatonin.^154^ Furthermore, both the oral administration of the amino acid and the indole lowered cytokine levels in aged birds.^162^

## Concluding Remarks

A variety of studies on serotonin neurotransmission indicate that, as a consequence of aging, a reduction in the density of serotonin receptors and marked disturbance in the 5-hydroxindole acetic/serotonin turnover and in the responses of the receptors/transporters to the hormonal regulation occur.^106^,^107^ Many of these alterations result in a decreased serotonin binding. Furthermore, the production of melatonin suffers a dramatic decline with age.^13^ These neurochemical changes may have etiologic implications in the altered behavior observed in old individuals and an underlying cause of several geriatric conditions, including the impaired sleep/wake cycle and immunosenescence. The evidence obtained in the ringdove and other animal models suggests that the supplemental administration of tryptophan, e.g. the inclusion of tryptophan-enriched food in the diet, might help to remediate the reduction in serotonin and melatonin that normally occurs as animals age, and be consequently beneficial in the treatment of sleep problems and alterations in the innate immune response.

## Figures and Tables

**Table 1.** Levels of melatonin in representative common vegetables and fruits measured using different methods by Dubbels et al.^175^ (1), Hattori et al.^176^ (2), and Badria^177^ (3).

**Common name**	**Scientific name**	**Melatonin (1)^a^**	**Melatonin (2)^b^**	**Melatonin (3)^c^**
Apple	*Malus domestica*	–	47.6 ± 3.1	16.1
Asparagus	*Asparagus officinalis*	–	9.5 ± 3.2	–
Banana	*Musa ensete*	–	–	65.5
Banana	*Musa sapientum*	46.6	–	–
Beetroot	*Beta vulgaris*	0.2	–	–
Cabbage	*Brassica oleracea var. capitata*	–	107.4 ± 7.3	30.9
Carrot	*Daucus carota*	–	55.3 ± 11.9	49.4
Corn	*Zea mays*	–	1366.1 ± 465.1	187.8
Cucumber	*Cucumis sativus*	8.6	24.6 ± 3.5	59.2
Garlic	*Allium sativum*	–	–	58.7
Ginger	*Zingiber officinale*	–	583.7 ± 50.3	142.3
Kiwi fruit	*Actinidia chinensis*	–	24.4 ± 1.7	–
Onion	*Allium cepa*	–	31.5 ± 4.8	29.9
Pineapple	*Ananas comosus*	–	36.2 ± 8.4	27.8
Pomegranate	*Punica granatum*	–	–	16.8
Radish	*Raphanus sativus*	–	–	75.8
Rice	*Oryza sativa*	–	1006.0 ± 58.5	149.8
Strawberry	*Fragaria magna*	–	12.4 ± 3.1	13.6
Tomato	*Lycopersicon esculentum*	50.6	32.2 ± 2.4	–
Tomato	*Lycopersicon pimpinellifolium*	11.2	–	30.2

a ng/100 g edible plant material (without peel). Levels measured by RIA and HPLC-MS.

b pg/g tissue. Levels quantified by RIA.

c ng/100 g. Levels measured by GC/MS analysis.
